# Supplementary material for: Similarity in Temporal Movement Patterns in Laying Hens Increases with Time and Social Association
Source: Animals (Basel). 2022 Feb 23;12(5):555. doi: 10.3390/ani12050555 (PMC8908832; doi:10.3390/ani12050555)
Supplement: Supplementary file 1 [file animals-12-00555-s001.zip › S1_Table.pdf]

S1 Table: Individual summary measures of activity and social network parameters. Trans: mean number of transitions, IN: proportion of time spent inside, Out: proportion of time spent outside (stone yard or free range), Entropy: mean sample entropy, Initiator: initiator score, Order out: mean ordinal number for entering the stone yard for the first time on a day, Order in: mean ordinal number for returning from the stone yard for the last time on a day, Days out: number of days the hen was observed at least once in either the stone yard or the free range area, PageRank: Page-Rank centrality in the social network (based on all observational days), Community: community membership base on Newman-Girven community partitioning. Mean numbers are always averaged over all observational days.

Pen	HenID	Trans	IN	Out	Entropy	Initiator	Order out	Order in	Days out	Between	Community
11	1	47.0	0.29	0.62	0.026	0.537	39	29	71	2.07	11.1
11	2	42.9	0.34	0.17	0.021	0.477	34	61	70	1.69	11.3
11	3	17.3	0.66	0.04	0.006	0.466	69	32	60	1.66	11.2
11	4	41.9	0.54	0.04	0.019	0.521	49	35	64	3.28	11.2
11	6	7.1	0.96	0.00	0.003	0.438	61	12	3	0.18	11.2
11	7	11.4	0.93	0.00	0.005	0.455	NA	NA	0	1.87	11.1
11	8	60.6	0.31	0.22	0.029	0.474	25	61	71	3.28	11.3
11	9	64.6	0.45	0.18	0.030	0.477	31	60	70	3.28	11.3
11	10	33.6	0.29	0.09	0.016	0.451	26	41	68	2.79	11.2
11	11	56.7	0.46	0.34	0.028	0.574	55	24	70	3.28	11.1
11	12	64.6	0.37	0.11	0.027	0.548	19	61	70	2.07	11.2
11	13	28.4	0.64	0.04	0.011	0.440	41	20	67	0.86	11.2
11	14	18.1	0.86	0.01	0.006	0.459	47	13	43	1.19	11.2
11	15	34.4	0.38	0.22	0.018	0.505	49	22	67	3.28	11.1
11	16	38.5	0.41	0.42	0.022	0.519	46	13	71	2.92	11.1
11	17	52.7	0.47	0.24	0.026	0.479	60	17	71	0.86	11.1
11	18	68.8	0.34	0.21	0.035	0.470	28	64	70	3.28	11.3
11	19	58.8	0.46	0.17	0.026	0.537	23	26	71	3.28	11.3
11	20	64.7	0.45	0.25	0.030	0.546	35	57	71	1.77	11.3
11	21	46.0	0.54	0.11	0.019	0.430	36	50	67	1.77	11.2
11	22	1.0	1.00	0.00	0.000	NA	NA	NA	0	0.00	11.4
11	23	32.5	0.35	0.08	0.016	0.483	52	37	68	0.59	11.2
11	24	44.3	0.46	0.28	0.021	0.532	37	46	71	0.86	11.3

Pen	HenID	Trans	IN	Out	Entropy	Initiator	Order out	Order in	Days out	Between	Community
11	25	5.2	0.96	0.00	0.002	0.474	NA	NA	0	0.08	11.2
11	26	13.2	0.87	0.03	0.004	0.522	35	11	58	0.49	11.2
11	27	40.6	0.46	0.37	0.021	0.492	55	25	70	3.28	11.1
11	28	76.0	0.31	0.30	0.040	0.539	39	60	71	3.28	11.3
11	29	67.4	0.25	0.32	0.037	0.511	22	52	71	0.86	11.3
11	30	34.5	0.78	0.07	0.008	0.476	68	32	69	3.28	11.2
11	500	73.1	0.58	0.19	0.031	0.566	63	18	68	2.02	11.1
11	501	56.5	0.36	0.07	0.028	0.560	55	66	68	2.02	11.2
11	503	44.1	0.33	0.09	0.024	0.536	79	23	64	3.28	11.1
11	504	35.6	0.41	0.11	0.016	0.569	40	38	70	3.28	11.2
11	505	23.1	0.86	0.03	0.007	0.522	86	41	50	1.89	11.1
11	506	76.2	0.58	0.09	0.031	0.538	37	61	71	3.28	11.2
11	507	80.3	0.39	0.21	0.038	0.545	40	63	70	3.28	11.3
11	508	25.5	0.28	0.23	0.015	0.596	45	18	63	1.77	11.1
11	509	49.1	0.39	0.24	0.026	0.551	45	55	69	3.19	11.3
11	511	52.5	0.23	0.55	0.029	0.574	54	35	70	3.28	11.1
11	512	38.2	0.42	0.11	0.020	0.521	78	41	67	2.02	11.1
11	513	26.8	0.67	0.04	0.010	0.535	65	47	60	2.95	11.2
11	515	63.3	0.38	0.13	0.030	0.531	32	52	70	2.02	11.2
11	516	56.1	0.32	0.33	0.031	0.547	55	18	68	2.02	11.1
11	518	15.1	0.65	0.07	0.007	0.491	83	18	40	1.89	11.1
11	519	60.5	0.40	0.13	0.030	0.525	33	44	67	2.07	11.3
11	520	22.8	0.47	0.07	0.012	0.519	69	13	43	1.84	11.1
11	521	24.6	0.57	0.06	0.012	0.524	51	21	29	2.07	11.1
11	522	43.0	0.71	0.03	0.015	0.522	52	19	64	3.28	11.2
11	523	49.8	0.55	0.11	0.017	0.545	28	52	71	1.77	11.3
11	524	20.8	0.62	0.03	0.010	0.498	73	14	44	0.76	11.1
11	526	63.1	0.60	0.05	0.025	0.512	35	30	68	2.02	11.2
11	527	16.7	0.45	0.04	0.009	0.507	49	4	39	1.75	11.1

Pen	HenID	Trans	IN	Out	Entropy	Initiator	Order out	Order in	Days out	Between	Community
11	528	36.4	0.37	0.34	0.021	0.522	53	9	71	2.92	11.1
11	529	76.8	0.35	0.26	0.037	0.519	43	63	71	2.07	11.3
11	531	1.0	1.00	0.00	0.000	NA	NA	NA	0	0.00	11.5
11	532	52.1	0.36	0.10	0.023	0.520	27	48	71	2.02	11.2
11	533	32.7	0.60	0.03	0.013	0.521	34	36	68	2.07	11.2
11	535	21.3	0.45	0.02	0.011	0.480	92	28	25	3.15	11.1
11	536	41.3	0.28	0.38	0.022	0.509	56	13	69	0.86	11.1
11	537	63.6	0.66	0.02	0.024	0.493	52	27	65	2.02	11.2
11	538	22.1	0.47	0.05	0.010	0.520	53	36	65	3.28	11.2
11	540	13.8	0.62	0.07	0.007	0.502	71	23	39	2.07	11.1
11	544	23.9	0.59	0.10	0.010	0.502	37	25	61	0.76	11.2
11	545	36.8	0.33	0.48	0.020	0.523	52	29	70	2.92	11.1
11	546	30.7	0.27	0.62	0.018	0.533	48	36	70	1.89	11.1
11	547	49.5	0.32	0.35	0.027	0.503	60	21	71	2.07	11.1
11	548	36.0	0.48	0.04	0.016	0.466	64	45	68	2.02	11.2
11	549	51.6	0.61	0.07	0.021	0.462	67	55	68	2.02	11.2
11	550	33.8	0.35	0.54	0.019	0.525	60	51	70	1.75	11.1
11	551	68.3	0.29	0.12	0.030	0.492	30	56	70	2.07	11.2
11	552	63.2	0.21	0.29	0.034	0.533	25	58	71	3.28	11.3
11	553	64.0	0.25	0.29	0.034	0.520	26	57	71	3.28	11.3
11	555	43.0	0.34	0.21	0.025	0.504	42	10	68	3.28	11.1
11	556	17.8	0.88	0.04	0.005	0.453	72	24	48	0.80	11.1
11	557	45.0	0.32	0.26	0.023	0.518	22	47	70	2.07	11.3
11	558	36.3	0.30	0.47	0.021	0.499	42	26	71	2.92	11.1
11	562	66.8	0.62	0.14	0.025	0.501	35	53	70	3.28	11.3
11	563	29.6	0.57	0.31	0.011	0.478	62	15	71	1.77	11.1
11	564	42.0	0.63	0.07	0.018	0.453	70	34	57	3.28	11.1
11	565	55.0	0.28	0.29	0.029	0.504	27	51	71	3.28	11.3
11	566	68.6	0.39	0.21	0.035	0.504	33	65	70	0.59	11.3

Pen	HenID	Trans	IN	Out	Entropy	Initiator	Order out	Order in	Days out	Between	Community
11	567	49.6	0.45	0.17	0.021	0.501	38	56	71	2.02	11.3
11	568	63.3	0.36	0.18	0.030	0.482	29	50	71	2.92	11.3
11	569	37.4	0.76	0.01	0.014	0.443	69	15	60	3.28	11.2
11	570	12.6	0.74	0.24	0.005	0.520	57	12	62	1.51	11.1
11	571	57.0	0.45	0.18	0.024	0.484	20	64	71	3.28	11.3
11	573	61.9	0.54	0.03	0.026	0.443	62	54	69	3.28	11.2
11	576	48.5	0.36	0.36	0.026	0.466	41	30	70	2.02	11.1
11	578	52.6	0.52	0.06	0.022	0.450	55	49	70	3.15	11.2
11	579	66.4	0.21	0.24	0.035	0.471	24	66	71	2.02	11.3
11	580	32.5	0.41	0.06	0.015	0.444	59	51	69	2.07	11.2
11	581	45.2	0.37	0.11	0.022	0.459	42	50	66	3.28	11.2
11	583	33.2	0.54	0.18	0.014	0.482	58	59	66	1.95	11.3
11	584	73.0	0.27	0.28	0.038	0.485	25	66	71	2.02	11.3
11	586	57.2	0.22	0.12	0.027	0.432	22	41	71	3.28	11.2
11	587	54.9	0.49	0.13	0.023	0.451	43	48	71	2.02	11.3
11	589	52.7	0.54	0.05	0.022	0.444	60	60	68	0.86	11.2
11	591	38.5	0.70	0.02	0.014	0.445	45	35	66	3.28	11.2
11	592	22.8	0.39	0.40	0.013	0.453	35	20	68	1.96	11.1
11	593	40.6	0.59	0.07	0.018	0.424	42	45	67	2.07	11.2
11	594	42.9	0.38	0.43	0.022	0.440	52	36	71	0.57	11.1
11	595	39.3	0.25	0.54	0.023	0.448	31	34	71	3.28	11.1
11	596	9.5	0.91	0.00	0.003	0.384	NA	NA	0	0.16	11.2
11	597	23.5	0.78	0.01	0.008	0.395	56	15	43	2.02	11.2
11	598	2.6	0.99	0.00	0.002	0.464	42	16	6	0.08	11.1
11	600	65.4	0.29	0.50	0.035	0.436	44	38	70	3.15	11.1
11	601	35.1	0.52	0.11	0.017	0.413	48	32	51	3.28	11.1
11	603	43.4	0.44	0.07	0.019	0.417	69	58	68	2.07	11.2
12	31	1.5	0.99	0.00	0.001	0.521	74	8	8	0.00	12.1
12	32	47.5	0.36	0.14	0.017	0.503	35	58	71	0.74	12.2

Pen	HenID	Trans	IN	Out	Entropy	Initiator	Order out	Order in	Days out	Between	Community
12	33	35.8	0.33	0.22	0.017	0.539	23	45	71	3.16	12.2
12	34	1.0	1.00	0.00	0.000	NA	NA	NA	0	0.00	12.3
12	35	19.9	0.58	0.06	0.007	0.582	73	44	62	0.26	12.1
12	36	28.4	0.51	0.05	0.011	0.501	43	11	69	0.56	12.2
12	37	24.8	0.40	0.04	0.011	0.513	88	50	60	0.26	12.1
12	38	39.4	0.30	0.15	0.018	0.478	36	68	72	6.09	12.2
12	39	1.0	1.00	0.00	0.000	NA	NA	NA	0	0.00	12.4
12	40	6.1	0.97	0.00	0.002	0.437	94	34	1	2.05	12.1
12	41	26.8	0.29	0.06	0.013	0.442	52	33	69	0.70	12.1
12	42	47.0	0.15	0.35	0.024	0.564	14	82	71	0.74	12.2
12	43	49.4	0.23	0.25	0.024	0.548	16	55	71	3.16	12.2
12	44	8.6	0.85	0.01	0.007	0.478	67	8	26	0.49	12.1
12	45	17.8	0.67	0.14	0.005	0.528	58	13	66	0.26	12.1
12	46	33.0	0.44	0.12	0.013	0.530	64	31	68	8.11	12.2
12	47	30.9	0.66	0.03	0.011	0.445	59	15	66	3.16	12.1
12	48	36.0	0.24	0.18	0.018	0.443	30	48	70	0.74	12.2
12	49	36.4	0.24	0.23	0.018	0.505	22	74	71	0.74	12.2
12	50	24.0	0.51	0.06	0.008	0.520	73	21	70	3.16	12.1
12	51	54.8	0.40	0.21	0.023	0.568	16	55	70	5.53	12.2
12	52	55.2	0.26	0.43	0.023	0.493	19	61	72	0.74	12.2
12	53	27.3	0.31	0.03	0.014	0.504	64	27	66	3.05	12.1
12	54	26.5	0.45	0.10	0.011	0.544	70	64	62	2.59	12.1
12	55	27.6	0.49	0.04	0.011	0.495	78	28	58	0.74	12.1
12	56	49.7	0.65	0.01	0.021	0.490	83	56	69	0.74	12.1
12	59	34.5	0.29	0.27	0.017	0.550	30	43	70	8.68	12.2
12	60	1.6	0.98	0.00	0.001	0.568	NA	NA	0	0.20	12.1
12	123	37.9	0.31	0.16	0.017	0.515	27	65	70	0.26	12.2
12	151	42.0	0.27	0.15	0.020	0.476	38	63	72	0.26	12.2
12	618	36.7	0.58	0.06	0.014	0.496	69	44	69	8.68	12.1

Pen	HenID	Trans	IN	Out	Entropy	Initiator	Order out	Order in	Days out	Between	Community
12	619	22.0	0.49	0.12	0.008	0.465	46	21	68	0.06	12.2
12	620	37.0	0.31	0.07	0.017	0.459	64	50	65	2.68	12.1
12	622	29.7	0.49	0.07	0.015	0.485	71	34	43	0.74	12.1
12	623	39.2	0.18	0.10	0.018	0.478	33	52	72	2.68	12.2
12	624	31.9	0.77	0.03	0.011	0.451	76	26	68	2.93	12.1
12	625	43.3	0.67	0.03	0.014	0.530	34	14	70	8.68	12.1
12	626	21.1	0.44	0.04	0.010	0.484	82	31	58	0.74	12.1
12	627	31.5	0.60	0.02	0.013	0.518	49	25	62	8.68	12.1
12	628	14.6	0.77	0.01	0.005	0.432	80	22	58	2.61	12.1
12	629	36.5	0.19	0.17	0.019	0.483	37	59	67	6.09	12.2
12	630	19.9	0.40	0.02	0.010	0.466	83	24	55	6.09	12.1
12	631	36.3	0.65	0.05	0.015	0.538	49	56	66	0.74	12.1
12	632	46.7	0.39	0.21	0.017	0.499	33	47	69	0.74	12.2
12	634	24.1	0.59	0.01	0.013	0.444	84	21	39	2.68	12.1
12	635	23.0	0.70	0.02	0.008	0.467	79	44	62	3.16	12.1
12	637	29.6	0.59	0.00	0.015	0.408	92	20	16	6.09	12.1
12	638	54.1	0.47	0.03	0.025	0.456	69	54	70	3.16	12.1
12	640	29.9	0.61	0.03	0.012	0.499	72	35	71	3.16	12.1
12	641	19.1	0.53	0.03	0.009	0.492	79	34	55	6.09	12.1
12	642	40.8	0.40	0.08	0.020	0.463	31	57	72	3.16	12.1
12	643	69.7	0.42	0.08	0.032	0.529	33	48	71	6.09	12.1
12	645	22.5	0.68	0.02	0.008	0.546	68	43	61	8.37	12.1
12	646	46.0	0.52	0.06	0.018	0.429	57	55	70	8.68	12.1
12	647	40.6	0.27	0.14	0.017	0.492	34	58	72	0.74	12.2
12	648	43.4	0.30	0.08	0.021	0.541	48	49	65	0.74	12.1
12	649	47.1	0.21	0.32	0.024	0.505	12	72	70	8.68	12.2
12	650	23.7	0.67	0.01	0.010	0.445	71	13	32	3.16	12.1
12	651	37.0	0.38	0.06	0.016	0.537	49	58	72	8.68	12.1
12	653	33.1	0.48	0.07	0.014	0.543	40	26	64	0.74	12.1

Pen	HenID	Trans	IN	Out	Entropy	Initiator	Order out	Order in	Days out	Between	Community
12	655	25.1	0.49	0.04	0.012	0.529	60	16	59	2.93	12.1
12	656	36.5	0.28	0.24	0.018	0.434	19	52	70	0.74	12.2
12	658	50.3	0.24	0.34	0.024	0.429	23	69	71	6.09	12.2
12	659	22.6	0.64	0.03	0.008	0.496	74	29	66	5.53	12.1
12	660	41.6	0.34	0.03	0.020	0.427	75	53	57	3.16	12.1
12	661	47.9	0.35	0.15	0.023	0.513	23	74	70	0.74	12.2
12	662	36.5	0.33	0.10	0.017	0.449	33	47	69	0.74	12.2
12	664	62.8	0.54	0.08	0.025	0.509	57	60	71	6.09	12.1
12	665	26.0	0.61	0.02	0.010	0.449	68	13	66	3.16	12.1
12	666	33.1	0.19	0.17	0.017	0.587	26	61	69	0.26	12.2
12	667	54.3	0.36	0.13	0.023	0.521	27	44	72	6.09	12.2
12	668	25.1	0.48	0.07	0.011	0.533	51	27	67	2.68	12.1
12	669	64.7	0.42	0.16	0.025	0.534	19	45	72	0.67	12.2
12	671	21.6	0.49	0.08	0.009	0.475	51	8	68	2.68	12.2
12	672	29.2	0.62	0.03	0.012	0.509	25	11	69	0.74	12.1
12	673	35.6	0.42	0.03	0.018	0.474	68	43	63	0.08	12.1
12	674	38.8	0.20	0.07	0.018	0.495	60	49	72	0.74	12.1
12	676	48.4	0.39	0.08	0.021	0.530	42	53	69	8.68	12.2
12	677	33.3	0.36	0.07	0.013	0.530	42	44	71	0.56	12.2
12	679	45.9	0.37	0.08	0.023	0.499	48	41	68	3.16	12.1
12	681	43.4	0.26	0.14	0.019	0.467	50	55	70	3.16	12.2
12	682	36.0	0.39	0.07	0.014	0.450	47	28	71	3.16	12.2
12	684	35.8	0.31	0.11	0.018	0.472	69	53	67	3.16	12.1
12	685	40.7	0.25	0.04	0.018	0.480	47	41	70	3.16	12.1
12	686	43.6	0.35	0.06	0.021	0.531	22	17	72	8.37	12.1
12	688	34.5	0.38	0.05	0.015	0.445	52	29	72	0.74	12.1
12	690	39.8	0.27	0.18	0.018	0.534	40	63	72	0.74	12.2
12	691	32.0	0.62	0.10	0.010	0.487	35	40	69	3.16	12.2
12	692	36.7	0.33	0.06	0.017	0.505	59	56	70	3.16	12.1

Pen	HenID	Trans	IN	Out	Entropy	Initiator	Order out	Order in	Days out	Between	Community
12	693	41.9	0.47	0.09	0.016	0.545	36	54	68	3.16	12.2
12	694	76.6	0.25	0.19	0.034	0.561	23	67	72	0.74	12.2
12	695	40.9	0.28	0.19	0.019	0.467	32	60	71	3.16	12.2
12	697	63.1	0.27	0.19	0.030	0.455	27	59	72	8.68	12.2
12	698	39.2	0.55	0.15	0.015	0.490	30	21	70	6.09	12.2
12	699	45.6	0.32	0.14	0.020	0.548	37	64	71	3.16	12.2
12	700	55.0	0.22	0.17	0.026	0.470	31	67	71	3.16	12.2
12	702	43.9	0.23	0.15	0.019	0.544	25	68	72	0.74	12.2
12	703	58.7	0.45	0.12	0.025	0.513	15	45	71	6.09	12.2
12	705	51.9	0.23	0.11	0.023	0.446	19	63	71	6.09	12.2
12	706	47.3	0.41	0.09	0.024	0.537	21	50	69	6.09	12.1
12	711	54.0	0.23	0.25	0.024	0.505	12	49	71	0.74	12.2
12	712	31.9	0.21	0.06	0.015	0.436	28	39	70	0.74	12.2
12	934	15.0	0.78	0.01	0.005	0.466	87	37	45	0.22	12.1
13	57	28.4	0.60	0.11	0.012	0.513	69	39	63	1.02	13.1
13	61	19.1	0.63	0.04	0.008	0.463	73	59	58	1.22	13.1
13	62	71.7	0.43	0.18	0.037	0.468	48	31	72	1.31	13.2
13	63	64.2	0.29	0.29	0.034	0.483	21	70	71	2.82	13.2
13	65	34.8	0.26	0.39	0.020	0.513	55	48	72	0.61	13.2
13	66	29.6	0.63	0.06	0.012	0.487	63	18	69	0.67	13.1
13	67	38.5	0.31	0.24	0.021	0.570	45	34	72	0.87	13.2
13	68	67.0	0.42	0.16	0.027	0.500	33	64	72	1.31	13.2
13	69	52.2	0.14	0.39	0.030	0.483	41	65	72	1.31	13.2
13	70	10.2	0.85	0.01	0.003	0.461	85	14	44	0.50	13.1
13	71	57.8	0.30	0.09	0.029	0.514	56	64	69	3.34	13.1
13	72	3.3	0.98	0.00	0.003	0.489	NA	NA	0	0.12	13.1
13	73	14.0	0.93	0.00	0.005	0.481	93	31	9	3.14	13.1
13	74	39.3	0.39	0.24	0.021	0.489	46	51	71	3.34	13.2
13	75	33.9	0.56	0.14	0.015	0.521	62	22	71	2.82	13.1

Pen	HenID	Trans	IN	Out	Entropy	Initiator	Order out	Order in	Days out	Between	Community
13	76	74.2	0.35	0.32	0.037	0.576	33	66	72	1.31	13.2
13	78	64.9	0.34	0.06	0.033	0.528	41	67	71	1.31	13.1
13	79	33.5	0.48	0.10	0.014	0.452	66	78	69	3.15	13.1
13	80	5.6	0.93	0.01	0.002	0.444	93	52	38	0.12	13.1
13	81	41.7	0.19	0.28	0.022	0.568	41	54	72	0.43	13.2
13	83	15.1	0.64	0.02	0.007	0.420	61	3	48	0.87	13.1
13	84	1.0	1.00	0.00	0.000	NA	NA	NA	0	0.00	13.3
13	85	22.1	0.73	0.14	0.008	0.552	79	64	54	2.82	13.2
13	86	20.4	0.30	0.13	0.010	0.490	38	35	60	1.31	13.1
13	88	51.2	0.36	0.17	0.027	0.550	35	39	71	1.31	13.2
13	89	5.2	0.92	0.01	0.003	0.493	77	5	24	0.30	13.1
13	90	28.7	0.46	0.02	0.015	0.377	89	33	57	1.31	13.1
13	104	35.0	0.56	0.06	0.012	0.461	62	53	70	1.31	13.1
13	113	40.7	0.23	0.25	0.025	0.538	44	41	72	1.31	13.2
13	153	17.8	0.77	0.05	0.005	0.449	84	34	71	2.44	13.1
13	720	20.1	0.78	0.09	0.006	0.574	48	8	69	0.87	13.2
13	721	1.0	1.00	0.00	0.000	NA	NA	NA	0	0.00	13.4
13	723	87.0	0.29	0.37	0.051	0.572	45	67	72	1.31	13.2
13	725	15.2	0.82	0.02	0.005	0.540	50	9	60	2.67	13.1
13	726	43.8	0.42	0.07	0.022	0.549	54	24	71	3.34	13.1
13	727	36.5	0.38	0.22	0.020	0.568	48	37	67	3.34	13.2
13	728	69.7	0.21	0.27	0.036	0.564	27	72	72	1.31	13.2
13	729	45.9	0.52	0.05	0.021	0.540	41	54	71	1.31	13.1
13	731	68.4	0.18	0.32	0.036	0.568	33	71	72	1.31	13.2
13	732	36.1	0.35	0.13	0.018	0.547	37	36	72	3.34	13.2
13	733	40.7	0.46	0.08	0.018	0.547	72	42	70	1.31	13.1
13	734	30.2	0.39	0.05	0.014	0.540	31	43	70	2.85	13.1
13	735	1.0	1.00	0.00	0.000	NA	NA	NA	0	0.00	13.5
13	738	57.0	0.53	0.14	0.026	0.549	40	45	71	3.34	13.2

Pen	HenID	Trans	IN	Out	Entropy	Initiator	Order out	Order in	Days out	Between	Community
13	740	52.6	0.40	0.25	0.025	0.548	37	43	72	3.34	13.2
13	741	50.0	0.45	0.03	0.025	0.525	63	46	66	3.34	13.1
13	742	62.5	0.30	0.22	0.032	0.542	42	70	72	3.34	13.2
13	744	65.2	0.28	0.18	0.031	0.542	28	60	72	3.34	13.2
13	745	63.2	0.23	0.14	0.034	0.533	22	46	72	0.84	13.2
13	746	50.5	0.62	0.05	0.020	0.513	68	55	71	0.84	13.1
13	747	23.8	0.51	0.06	0.012	0.488	72	15	49	2.85	13.1
13	748	45.2	0.43	0.10	0.021	0.535	39	54	70	3.34	13.1
13	750	73.8	0.32	0.29	0.038	0.519	48	70	72	3.34	13.2
13	752	50.0	0.45	0.21	0.026	0.527	51	46	70	1.31	13.2
13	753	39.3	0.36	0.07	0.019	0.499	35	38	70	1.31	13.1
13	754	29.8	0.50	0.07	0.014	0.503	80	61	56	3.34	13.1
13	758	41.0	0.20	0.33	0.024	0.527	39	56	72	2.36	13.2
13	759	58.8	0.31	0.23	0.029	0.530	26	66	72	3.34	13.2
13	760	75.6	0.36	0.19	0.035	0.484	16	71	72	3.34	13.2
13	761	43.8	0.30	0.09	0.021	0.498	38	51	71	1.31	13.2
13	762	85.9	0.23	0.22	0.048	0.527	28	66	72	1.31	13.2
13	763	35.6	0.43	0.29	0.016	0.531	48	60	71	1.02	13.2
13	764	39.8	0.47	0.05	0.019	0.478	69	50	71	3.34	13.1
13	765	41.7	0.53	0.04	0.019	0.478	66	23	72	3.34	13.1
13	766	21.6	0.53	0.01	0.010	0.458	76	17	68	0.84	13.1
13	767	28.6	0.61	0.02	0.013	0.469	72	15	55	3.34	13.1
13	768	41.6	0.25	0.21	0.025	0.518	43	38	71	0.84	13.2
13	769	24.7	0.70	0.06	0.008	0.532	80	29	71	2.82	13.1
13	770	67.7	0.39	0.08	0.033	0.508	41	40	70	1.31	13.1
13	772	46.3	0.30	0.06	0.023	0.468	32	48	72	3.34	13.1
13	774	47.0	0.33	0.22	0.021	0.509	30	68	72	1.31	13.2
13	776	47.0	0.25	0.05	0.024	0.475	43	39	71	1.31	13.1
13	777	54.7	0.50	0.12	0.024	0.495	44	48	71	2.85	13.2

Pen	HenID	Trans	IN	Out	Entropy	Initiator	Order out	Order in	Days out	Between	Community
13	779	39.9	0.54	0.01	0.020	0.457	54	27	56	2.82	13.1
13	780	57.9	0.44	0.04	0.027	0.475	53	59	71	1.31	13.1
13	781	20.6	0.81	0.04	0.005	0.473	80	28	70	2.60	13.1
13	782	34.6	0.27	0.03	0.017	0.484	38	46	70	3.34	13.1
13	784	63.0	0.39	0.06	0.030	0.478	44	54	72	3.34	13.1
13	785	50.7	0.53	0.04	0.023	0.453	69	30	69	2.85	13.1
13	786	47.3	0.30	0.07	0.022	0.486	31	64	72	1.31	13.1
13	789	57.8	0.30	0.28	0.033	0.493	37	45	72	1.09	13.2
13	790	69.5	0.24	0.22	0.037	0.494	48	59	72	0.87	13.2
13	791	45.2	0.22	0.12	0.023	0.481	40	72	71	1.31	13.2
13	792	69.3	0.20	0.38	0.044	0.489	49	49	72	1.31	13.2
13	793	38.8	0.39	0.13	0.018	0.466	36	30	72	2.36	13.2
13	795	61.0	0.62	0.03	0.025	0.443	74	64	70	2.82	13.1
13	796	20.0	0.82	0.01	0.007	0.426	79	28	52	2.67	13.1
13	797	70.4	0.25	0.19	0.036	0.459	26	65	72	3.34	13.2
13	798	42.5	0.24	0.09	0.021	0.461	33	41	70	1.31	13.1
13	799	19.4	0.91	0.00	0.007	0.418	93	23	1	2.82	13.1
13	800	20.8	0.67	0.13	0.008	0.468	85	56	65	2.82	13.1
13	801	66.2	0.46	0.07	0.033	0.448	48	34	70	3.34	13.1
13	802	44.1	0.39	0.12	0.024	0.447	27	45	67	1.31	13.1
13	805	66.9	0.30	0.30	0.036	0.491	35	62	72	1.31	13.2
13	806	12.7	0.76	0.05	0.004	0.443	73	17	65	0.59	13.1
13	810	52.5	0.39	0.08	0.024	0.447	34	48	70	3.34	13.1
13	811	29.1	0.67	0.08	0.009	0.476	82	56	67	3.34	13.1
13	812	82.9	0.21	0.29	0.043	0.477	20	71	72	1.31	13.2
13	813	57.5	0.35	0.12	0.029	0.451	34	69	70	3.34	13.2
13	814	36.7	0.47	0.02	0.017	0.423	66	29	70	1.31	13.1
13	815	40.7	0.47	0.09	0.019	0.439	37	35	72	3.34	13.1
13	817	18.0	0.61	0.11	0.014	0.453	41	43	35	2.88	13.2

Pen	HenID	Trans	IN	Out	Entropy	Initiator	Order out	Order in	Days out	Between	Community
13	819	45.6	0.33	0.15	0.022	0.444	41	41	72	1.31	13.1
13	821	39.6	0.56	0.04	0.017	0.418	40	40	71	3.07	13.1
13	822	42.0	0.46	0.03	0.020	0.419	72	47	63	3.34	13.1
13	823	30.6	0.66	0.06	0.014	0.415	53	8	59	2.85	13.1
14	91	48.6	0.38	0.31	0.022	0.532	8	57	72	2.39	14.1
14	92	5.4	0.88	0.03	0.003	0.459	65	2	25	1.25	14.3
14	93	35.3	0.62	0.08	0.013	0.499	40	25	67	2.79	14.1
14	94	31.6	0.56	0.02	0.013	0.527	55	34	68	3.17	14.3
14	95	44.2	0.36	0.45	0.027	0.513	44	30	72	3.17	14.2
14	97	1.0	1.00	0.00	0.001	0.625	NA	NA	0	0.00	14.1
14	98	1.0	1.00	0.00	0.000	NA	NA	NA	0	0.00	14.4
14	99	41.6	0.40	0.50	0.023	0.561	47	24	72	3.04	14.2
14	100	77.8	0.29	0.30	0.037	0.496	14	59	72	1.74	14.1
14	101	9.2	0.90	0.00	0.004	0.446	71	10	17	1.77	14.3
14	102	51.6	0.33	0.16	0.025	0.516	28	57	72	2.24	14.1
14	103	47.8	0.28	0.06	0.023	0.469	29	38	72	2.88	14.1
14	105	28.6	0.76	0.04	0.010	0.434	53	15	53	2.02	14.3
14	106	58.8	0.41	0.20	0.025	0.559	18	52	72	2.24	14.1
14	107	6.6	0.91	0.01	0.003	0.399	71	5	16	1.45	14.3
14	108	69.0	0.39	0.07	0.035	0.542	35	47	71	3.17	14.1
14	109	78.8	0.37	0.16	0.032	0.479	25	52	72	2.64	14.1
14	110	21.8	0.48	0.13	0.013	0.505	74	30	37	2.58	14.2
14	111	24.0	0.58	0.08	0.010	0.545	42	24	63	1.56	14.1
14	112	43.6	0.33	0.12	0.020	0.475	18	35	72	3.17	14.1
14	114	56.1	0.51	0.21	0.024	0.477	22	43	68	2.29	14.1
14	115	41.8	0.47	0.28	0.017	0.552	20	31	71	1.36	14.1
14	117	9.3	0.86	0.03	0.003	0.498	69	17	52	1.90	14.3
14	119	3.4	0.95	0.00	0.002	0.496	69	7	13	0.22	14.3
14	121	52.3	0.30	0.32	0.025	0.484	6	54	72	1.42	14.1

Pen	HenID	Trans	IN	Out	Entropy	Initiator	Order out	Order in	Days out	Between	Community
14	122	32.1	0.45	0.46	0.019	0.535	55	18	72	2.96	14.2
14	124	35.1	0.25	0.72	0.018	0.534	53	43	72	2.24	14.2
14	150	31.1	0.41	0.03	0.018	0.435	72	18	23	3.17	14.2
14	152	37.6	0.47	0.42	0.020	0.567	59	27	72	2.88	14.2
14	154	55.6	0.56	0.20	0.029	0.484	50	9	72	3.17	14.2
14	836	18.6	0.63	0.02	0.008	0.401	73	33	51	2.81	14.3
14	839	11.6	0.90	0.03	0.003	0.496	66	19	50	1.04	14.3
14	840	52.5	0.51	0.12	0.024	0.521	21	41	66	2.58	14.1
14	841	23.2	0.67	0.04	0.010	0.429	68	32	50	2.26	14.3
14	843	6.8	0.93	0.00	0.003	0.405	NA	NA	0	0.39	14.2
14	844	25.2	0.54	0.22	0.014	0.522	54	8	61	3.17	14.2
14	845	4.6	0.87	0.00	0.002	0.401	NA	NA	1	0.46	14.3
14	846	7.2	0.77	0.04	0.004	0.520	43	9	35	0.82	14.2
14	847	37.5	0.38	0.33	0.023	0.431	51	10	70	3.17	14.2
14	848	52.2	0.37	0.09	0.024	0.516	26	49	67	3.17	14.1
14	849	28.6	0.71	0.11	0.012	0.490	73	24	34	2.05	14.2
14	850	6.2	0.83	0.00	0.003	0.480	84	17	4	0.60	14.3
14	851	1.0	1.00	0.00	0.000	NA	NA	NA	0	0.00	14.5
14	852	30.8	0.51	0.27	0.016	0.470	63	27	70	2.79	14.2
14	853	29.0	0.68	0.17	0.012	0.439	72	35	63	3.17	14.2
14	854	38.2	0.35	0.23	0.018	0.544	21	38	72	1.21	14.1
14	855	31.1	0.31	0.16	0.016	0.555	15	37	70	2.39	14.1
14	856	27.4	0.48	0.33	0.014	0.468	53	14	71	1.39	14.2
14	858	35.6	0.38	0.32	0.022	0.466	64	44	58	2.28	14.2
14	859	73.8	0.32	0.16	0.033	0.476	14	58	72	3.17	14.1
14	860	31.8	0.44	0.36	0.016	0.544	48	21	62	3.17	14.2
14	861	44.4	0.60	0.07	0.018	0.452	17	52	64	3.17	14.1
14	862	43.3	0.67	0.11	0.014	0.478	21	35	66	3.17	14.1
14	863	59.6	0.43	0.22	0.022	0.551	33	55	72	1.41	14.1

Pen	HenID	Trans	IN	Out	Entropy	Initiator	Order out	Order in	Days out	Between	Community
14	865	45.8	0.61	0.12	0.016	0.517	47	36	72	2.39	14.1
14	867	26.3	0.70	0.04	0.010	0.505	39	26	68	2.24	14.3
14	868	70.2	0.28	0.19	0.034	0.514	7	58	72	97.68	14.1
14	869	34.5	0.44	0.44	0.016	0.512	52	34	71	1.68	14.2
14	870	33.3	0.62	0.01	0.013	0.471	61	45	65	2.24	14.3
14	871	78.2	0.45	0.08	0.034	0.506	25	58	72	3.17	14.1
14	872	33.6	0.39	0.05	0.016	0.563	39	32	58	2.24	14.1
14	873	19.9	0.29	0.05	0.010	0.445	31	25	66	1.29	14.3
14	874	53.5	0.49	0.07	0.021	0.462	23	39	71	2.24	14.1
14	875	50.0	0.52	0.02	0.022	0.426	50	45	70	3.17	14.3
14	876	33.7	0.39	0.30	0.020	0.536	66	12	63	3.17	14.2
14	878	41.1	0.34	0.08	0.019	0.489	16	48	72	2.58	14.1
14	879	45.6	0.50	0.12	0.024	0.511	53	8	62	2.95	14.2
14	880	46.5	0.53	0.33	0.024	0.495	49	20	72	2.88	14.2
14	881	35.6	0.72	0.04	0.011	0.458	63	55	70	2.58	14.3
14	882	45.0	0.42	0.10	0.019	0.540	44	58	71	3.17	14.1
14	884	22.4	0.60	0.02	0.012	0.427	43	6	26	3.17	14.2
14	885	10.9	0.57	0.00	0.006	0.448	NA	NA	3	2.22	14.2
14	886	39.1	0.54	0.07	0.014	0.530	26	37	70	2.79	14.1
14	888	33.5	0.38	0.27	0.020	0.549	60	29	62	3.17	14.2
14	891	48.0	0.48	0.06	0.020	0.530	24	46	72	2.24	14.1
14	892	23.5	0.29	0.31	0.015	0.485	61	30	70	3.17	14.2
14	893	63.0	0.25	0.09	0.031	0.443	53	63	72	3.17	14.1
14	894	4.6	0.86	0.02	0.003	0.575	79	31	6	0.65	14.2
14	895	52.2	0.33	0.05	0.024	0.464	26	36	71	3.17	14.1
14	896	59.1	0.38	0.13	0.026	0.486	18	56	72	3.17	14.1
14	898	42.0	0.33	0.07	0.018	0.532	37	48	72	3.17	14.1
14	899	53.0	0.36	0.12	0.023	0.497	23	64	71	3.17	14.1
14	900	50.8	0.42	0.22	0.031	0.455	55	20	65	3.17	14.2

Pen	HenID	Trans	IN	Out	Entropy	Initiator	Order out	Order in	Days out	Between	Community
14	902	37.2	0.52	0.27	0.021	0.528	56	26	67	2.24	14.2
14	904	24.5	0.47	0.41	0.013	0.539	51	31	68	1.97	14.2
14	905	76.8	0.35	0.26	0.037	0.461	7	59	72	3.17	14.1
14	907	46.2	0.41	0.06	0.022	0.480	45	35	72	2.24	14.1
14	909	35.5	0.63	0.03	0.017	0.425	65	16	16	2.26	14.2
14	910	17.1	0.58	0.05	0.006	0.496	46	16	67	2.95	14.1
14	912	43.8	0.45	0.05	0.019	0.542	31	37	72	3.17	14.1
14	913	47.8	0.45	0.28	0.026	0.525	58	33	71	3.17	14.2
14	917	55.7	0.31	0.08	0.026	0.519	15	33	72	3.17	14.1
14	918	53.1	0.29	0.14	0.024	0.542	33	61	72	2.64	14.1
14	919	1.0	1.00	0.00	0.000	NA	NA	NA	0	0.00	14.6
14	921	61.4	0.32	0.10	0.027	0.524	21	49	72	103.17	14.1
14	924	34.3	0.37	0.06	0.014	0.529	35	33	72	3.17	14.1
14	926	48.7	0.30	0.33	0.032	0.457	46	23	65	3.17	14.2
14	928	31.8	0.29	0.39	0.020	0.535	40	21	67	1.37	14.2
14	929	28.6	0.31	0.15	0.017	0.424	59	11	51	2.24	14.2
14	930	45.9	0.49	0.27	0.025	0.473	49	17	69	2.58	14.2
14	931	23.8	0.41	0.30	0.014	0.427	45	11	70	2.14	14.2
14	932	35.6	0.32	0.41	0.020	0.570	40	47	63	2.42	14.2
14	933	63.4	0.31	0.62	0.036	0.541	45	45	72	3.17	14.2
14	935	81.8	0.46	0.24	0.033	0.523	23	62	71	2.64	14.1
