# Supplementary material for: Similarity in Temporal Movement Patterns in Laying Hens Increases with Time and Social Association
Source: Animals (Basel). 2022 Feb 23;12(5):555. doi: 10.3390/ani12050555 (PMC8908832; doi:10.3390/ani12050555)
Supplement: Supplementary file 1 [file animals-12-00555-s001.zip › S2_Table_revised.pdf]

S2 Table: Time gaps between transitions of two hens from one area to the next. Time bound (s) of the first 25% quartile. Observed value (Obs.) and expected value (Exp.) based on simulated sampling assuming randomly dispersed and independent transitions of hens. For calculating expected values under the null assumption, we randomly sampled transition times from a uniform distribution for the time span from the first to the last transition. Arrows indicate the direction of transitions; IN: indoors, WG: wintergarden, SY: stone yard, FR: free range. KS-Test: test statistics for the Kolmogorov-Smirnov test for distribution fit ($p<0.001$ in all cases).

	Pen 11			Pen 12			Pen 13			Pen 14		
	Obs	Exp	KS-test	Obs	Exp	KS-test	Obs	Exp	KS-test	Obs	Exp	KS-test
IN → WG	3	6	0.1167	4	7	0.1238	4	6	0.1076	4	7	0.1294
WG → SY	6	12	0.1285	5	8	0.1131	5	8	0.1043	7	12	0.1108
SY → FR	3	10	0.2302	13	40	0.1759	6	15	0.1521	4	12	0.2023
FR → SY	1	10	0.3308	7	44	0.2302	4	16	0.2152	3	14	0.2505
SY → WG	4	13	0.1907	3	8	0.1794	4	8	0.1535	6	14	0.1520
WG → IN	3	6	0.1504	3	7	0.1487	3	7	0.1452	3	7	0.1534
