# Supplementary material for: Similarity in Temporal Movement Patterns in Laying Hens Increases with Time and Social Association
Source: Animals (Basel). 2022 Feb 23;12(5):555. doi: 10.3390/ani12050555 (PMC8908832; doi:10.3390/ani12050555)
Supplement: Supplementary file 1 [file animals-12-00555-s001.zip › S3_Table.pdf]

S3 Table: Summary table of the cluster characteristics per pen.

Variable	Cluster	Pen 11			Pen 12			Pen 13			Pen 14		
		mean (sd)	median	range	mean	median	range	mean	median	range	mean	median	range
total number of transitions	1	13.85 (14.47)	9	1; 91	9.04 (14.87)	1	1; 90	55.63 (28.57)	51	1; 159	45.07 (24.58)	41	1; 202
	2	50.16 (24.47)	47	1; 155	27.61 (19.16)	25	1; 103	38.60 (24.24)	35	1; 154	7.10 (10.63)	3	1; 79
	3	50.73 (25.46)	48	1; 152	36.99 (20.74)	34	1; 176	51.12 (25.79)	48	1; 160	28.68 (23.15)	24	1; 140
	4	36.23 (24.24)	31	1; 145	43.78 (21.74)	41	1; 158	15.44 (18.12)	9	1; 130	44.40 (24.24)	41	1; 173
total time spent inside [min]	1	311.80 (137.75)	371.25	0; 525	364.95 (112.58)	410	71.27; 525	89.49 (86.44)	68.44	0; 470	154.82 (88.17)	137.4	0; 484.65
	2	110.73 (67.69)	94.8	0; 500	240.18 (123.94)	231.84	0; 525	203.04 (110.19)	184.65	0; 505	358.22 (114.81)	399.58	0; 525
	3	146.25 (84.87)	128.63	0; 514.23	178.62 (97.10)	160.82	0; 515	122.69 (83.45)	102.43	0; 495.48	238.90 (121.51)	235.5	0; 524.7
	4	220.32 (117.28)	212.78	0; 525	126.86 (73.43)	110.38	0; 474.82	307.49 (144.33)	366.92	0; 525	119.93 (72.80)	106.12	0; 459.15
total time spent in WG [min]	1	55.24 (68.64)	27.7	0; 317.02	48.79 (80.55)	0	0; 312.55	128.50 (68.95)	123.68	0; 364.92	148.30 (87.65)	142.58	0; 398.2
	2	94.87 (75.51)	82.79	0; 405.83	147.30 (92.15)	152.48	0; 384.43	151.25 (85.26)	152.07	0; 483.1	41.88 (68.20)	8.95	0; 348.5
	3	132.188 (78.29)	127.82	0; 422.47	195.98 (84.14)	201.03	0; 417.02	152.15 (75.36)	149.83	0; 392.03	114.05 (90.25)	93.57	0; 444.1
	4	124.18 (82.71)	117.96	0; 416.77	203.72 (75.00)	208.53	0; 390.35	60.05 (74.29)	35.48	0; 381.03	86.13 (75.63)	71.47	0; 386.53
total time spent in SY [min]	1	6.36 (16.96)	0	0; 127.97	4.68 (18.81)	0	0; 228.87	37.13 (28.35)	32.79	0; 177.23	19.75 (22.58)	13.33	0; 256.7
	2	40.82 (40.36)	31.04	0; 272.23	25.94 (38.35)	8.73	0; 265.15	13.78 (16.80)	7.93	0; 192	1.70 (7.73)	0	0; 76.7
	3	26.30 (31.05)	17.58	0; 313.8	32.16 (43.99)	15.24	0; 318.78	29.60 (26.22)	23.22	0; 266.22	10.37 (18.60)	1.58	0; 142.58
	4	16.41 (34.83)	4.48	0; 375.43	61.17 (62.28)	39.27	0; 299.3	7.97 (14.96)	0.82	0; 115.72	30.16 (31.14)	22.43	0; 245
total time spent in FR [min]	1	5.94 (19.19)	0	0; 168.13	1.18 (7.53)	0	0; 80.55	30.11 (31.10)	22.46	0; 200.1	18.82 (31.76)	2.58	0; 248.18
	2	56.83 (52.39)	43.46	0; 301.15	2.97 (11.79)	0	0; 186.82	7.01 (15.33)	0	0; 183.32	3.53 (16.81)	0	0; 193.43
	3	26.89 (36.36)	11.9	0; 233.12	3.75 (12.13)	0	0; 238.58	19.25 (25.24)	9.85	0; 217.42	14.00 (30.51)	0	0; 219.53
	4	12.30 (27.42)	0.93	0; 223.98	9.99 (24.82)	0	0; 237.12	6.16 (17.07)	0	0; 156	56.09 (54.69)	41.53	0; 365.4
proportion of the time spent outside the barn	1	0.04 (0.11)	0	0; 0.76	0.02 (0.05)	0	0; 0.63	0.27 (0.18)	0.24	0; 1	0.14 (0.17)	0.07	0; 1
	2	0.34 (0.24)	0.31	0; 1	0.07 (0.10)	0.03	0; 0.61	0.07 (0.09)	0.03	0; 1	0.02 (0.10)	0	0; 1
	3	0.18 (0.19)	0.12	0; 1	0.09 (0.12)	0.05	0; 1	0.17 (0.16)	0.14	0; 1	0.09 (0.16)	0.01	0; 1
	4	0.1 (0.18)	0.02	0; 1	0.18 (0.17)	0.14	0; 0.97	0.05 (0.11)	0	0; 0.76	0.32 (0.25)	0.27	0; 1
order appearing in SY	1	45.12 (24.43)	45	1; 92	53.36 (25.86)	56.5	1; 97	39.57 (25.18)	41	1; 95	36.18 (21.81)	34	1; 87
	2	41.67 (23.06)	42	1; 98	51.41 (26.78)	54	1; 96	52.48 (27.38)	55	1; 100	56.95 (20.05)	64	13; 84
	3	38.95 (24.11)	37	1; 97	48.34 (26.36)	49	1; 97	41.06 (25.25)	39	1; 97	46.22 (23.07)	47	1; 87
	4	48.63 (26.02)	52	1; 97	33.81 (24.08)	29	1; 91	65.18 (26.26)	72.5	1; 99	37.51 (22.30)	39	1; 88
order leaving SY	1	32.01 (22.58)	27	1; 85	34.47 (27.79)	27	1; 87	54.08 (25.46)	55	1; 100	40.74 (22.16)	41	1; 87
	2	42.3 (25.06)	42	1; 98	41.11 (27.93)	37	1; 97	42.76 (27.38)	40	1; 99	27 (21.80)	18	1; 87
	3	44.75 (24.49)	45	1; 97	45.16 (26.43)	44	1; 97	51.48 (26.34)	53	1; 99	35.44 (22.85)	33	1; 86
	4	41.43 (24.84)	40	1; 97	52.79 (25.52)	55	1; 96	40.88 (28.71)	38	1; 99	38.60 (22.79)	39	1; 88
