# Supplementary material for: Similarity in Temporal Movement Patterns in Laying Hens Increases with Time and Social Association
Source: Animals (Basel). 2022 Feb 23;12(5):555. doi: 10.3390/ani12050555 (PMC8908832; doi:10.3390/ani12050555)
Supplement: Supplementary file 1 [file animals-12-00555-s001.zip › S4_Table.pdf]

S4 Table: Overall descriptors of the association networks derived from 72 observation days. Co-occurrences gives the total number of recorded co-occurrences of two birds at an antenna over the 72 observational days, density gives for the resulting network the proportion of observed links between hens based on co-occurrence, assortativity gives the network-wide assortativity index and community count is the number of sub-communities based on the Newman-Girvan community detection algorithm.

	Pen 11	Pen 12	Pen 13	Pen 14
Co-occurrences	134060	95219	114310	101334
Density	0.94	0.93	0.92	0.90
Assortativity	0.093	0.084	0.109	0.121
Community count	5	4	5	6
