# Supplementary figures and images for: Similarity in Temporal Movement Patterns in Laying Hens Increases with Time and Social Association
Source: Animals (Basel). 2022 Feb 23;12(5):555. doi: 10.3390/ani12050555 (PMC8908832; doi:10.3390/ani12050555)

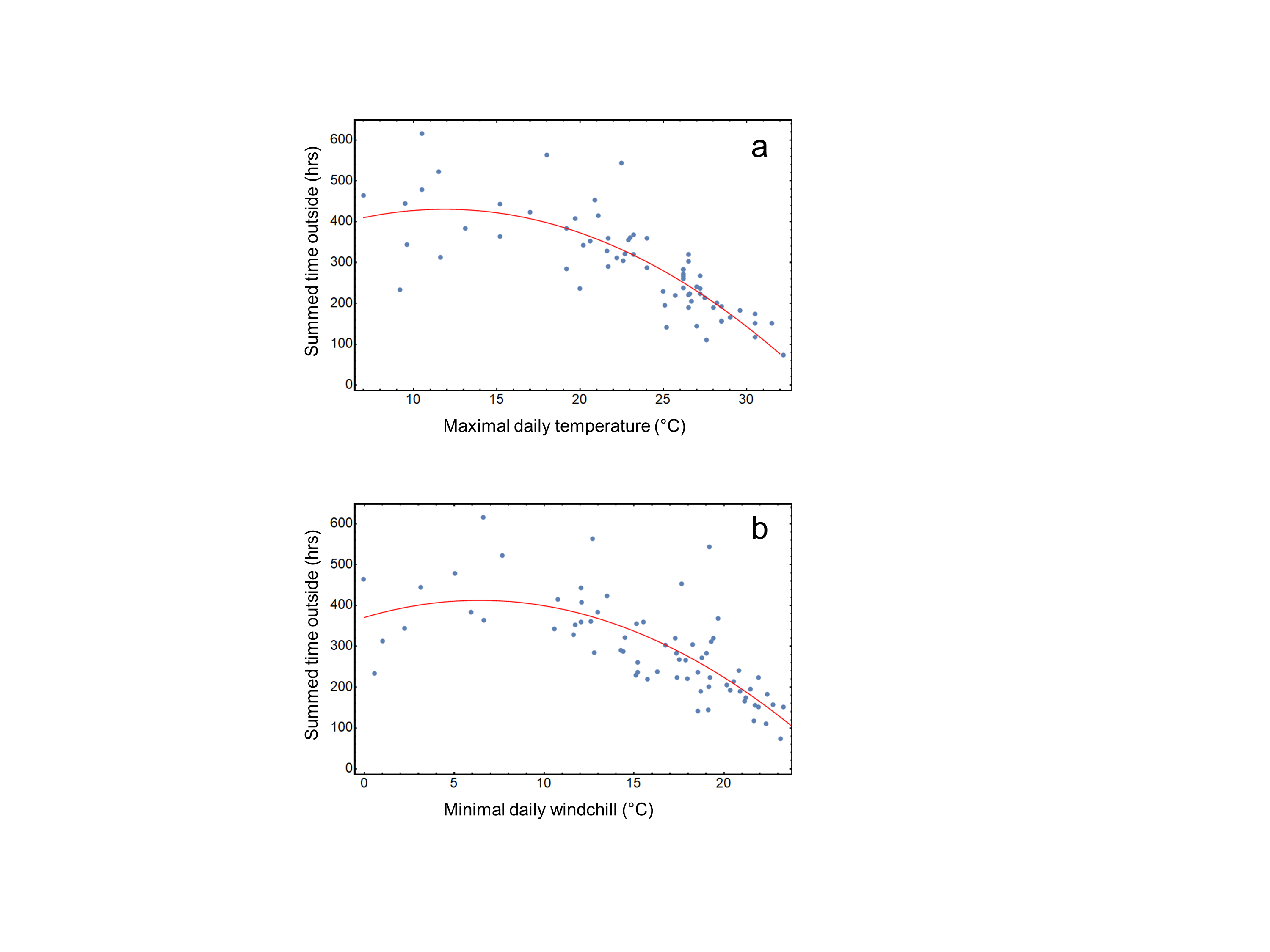

Supplement: Supplementary file 1 [file animals-12-00555-s001.zip › S1 Fig.tiff]

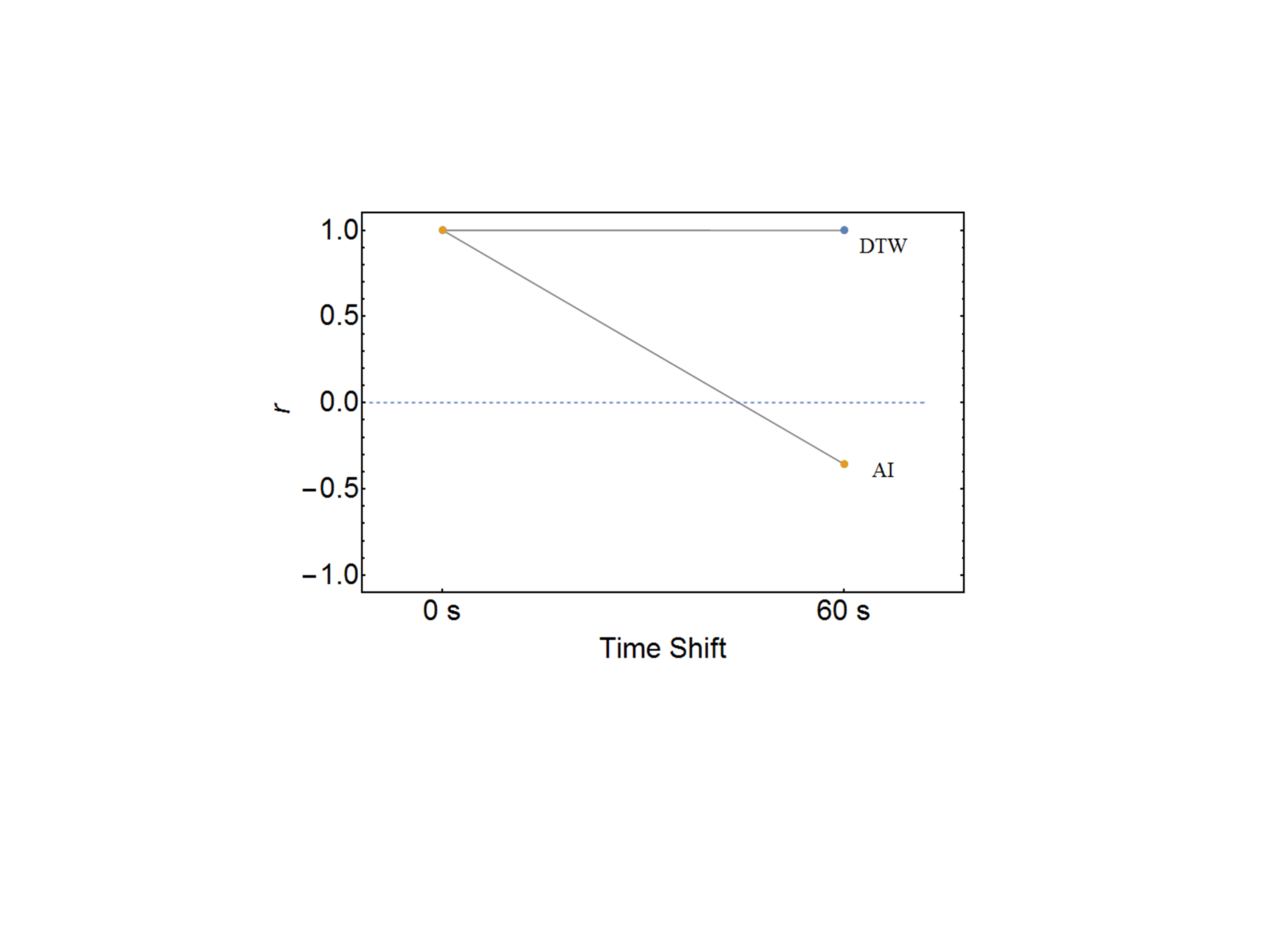

Supplement: Supplementary file 1 [file animals-12-00555-s001.zip › S2 Fig.tiff]

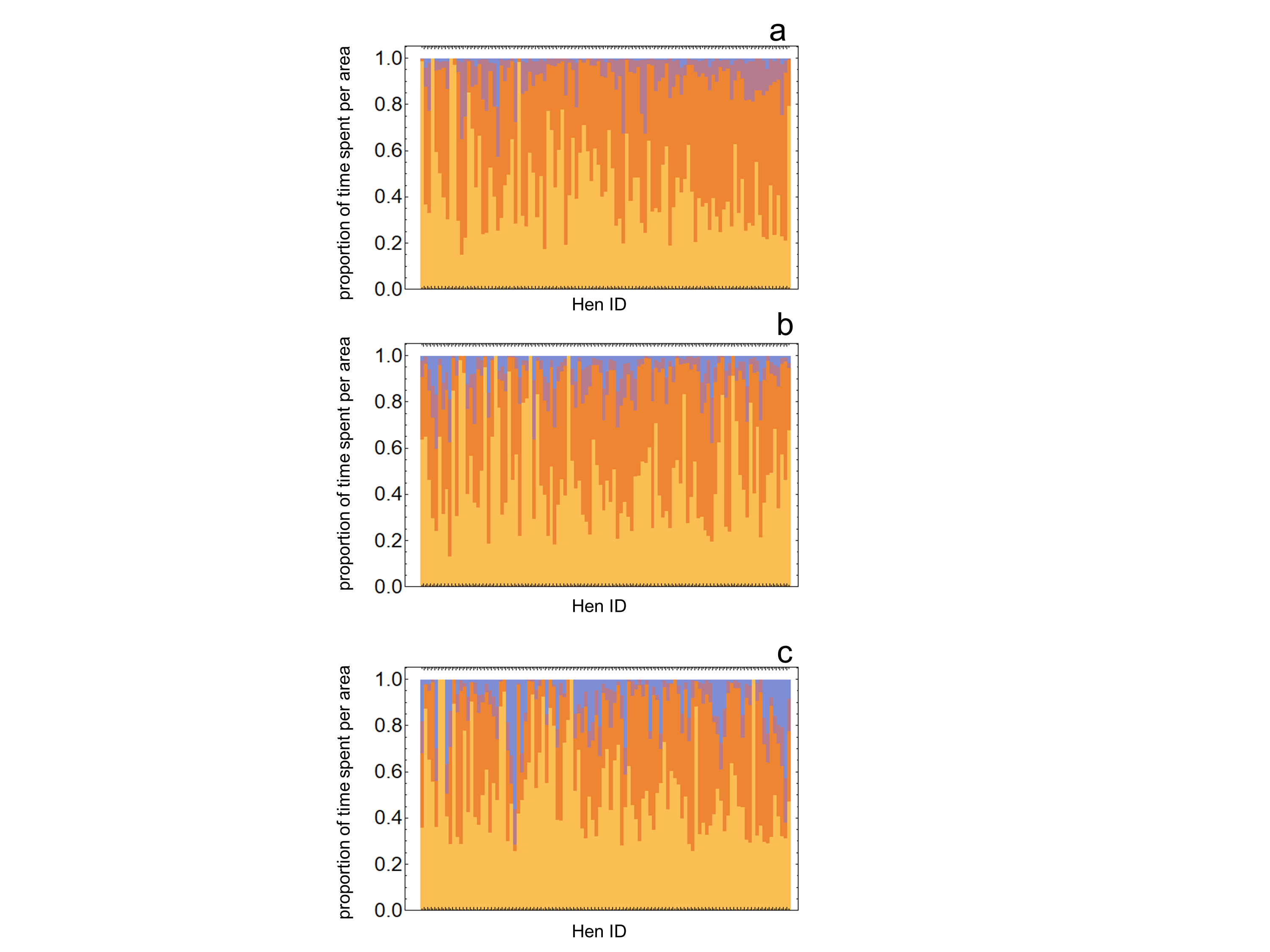

Supplement: Supplementary file 1 [file animals-12-00555-s001.zip › S3 Fig_v1.tiff]

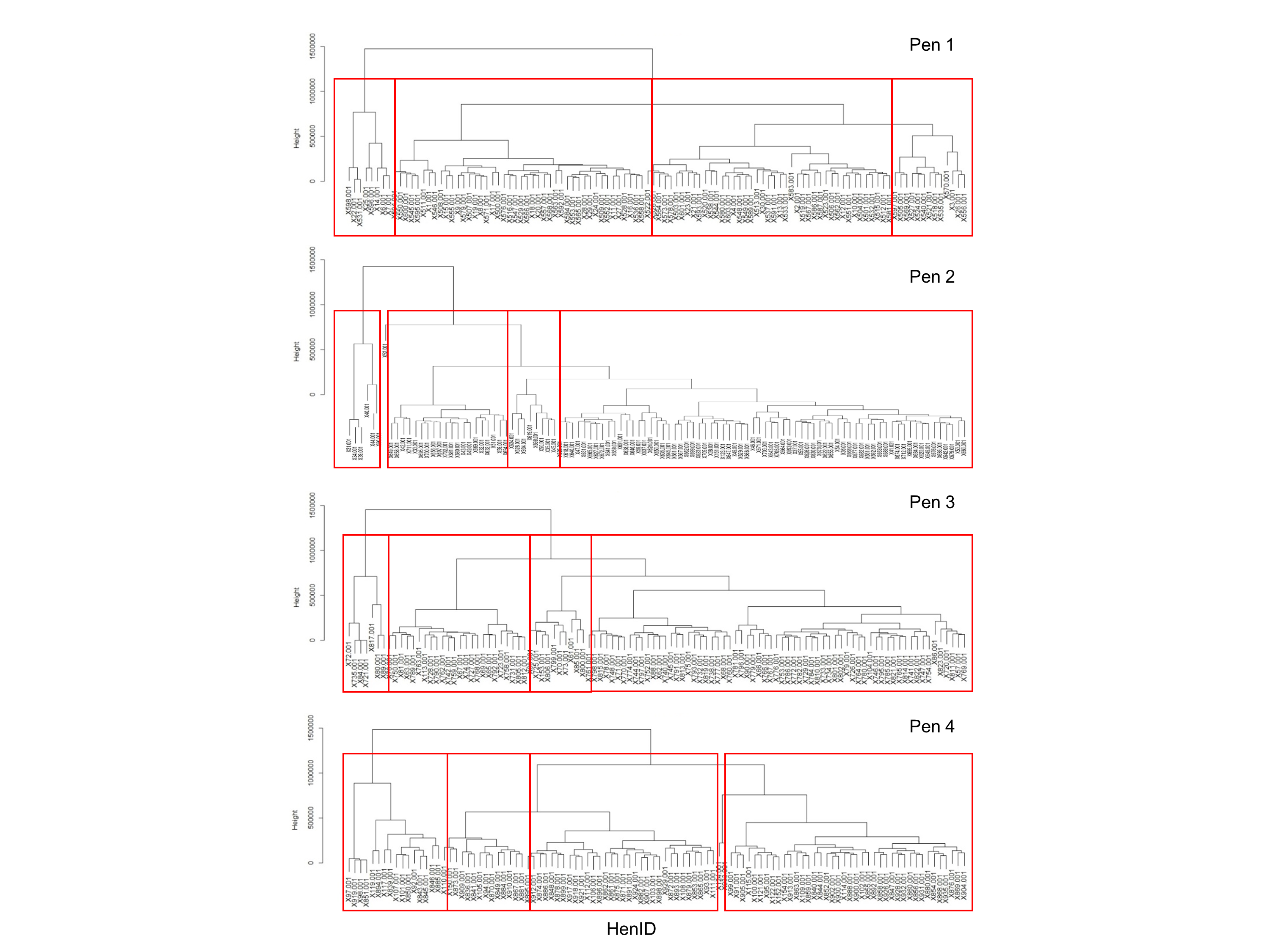

Supplement: Supplementary file 1 [file animals-12-00555-s001.zip › S4 Fig_v1.tiff]

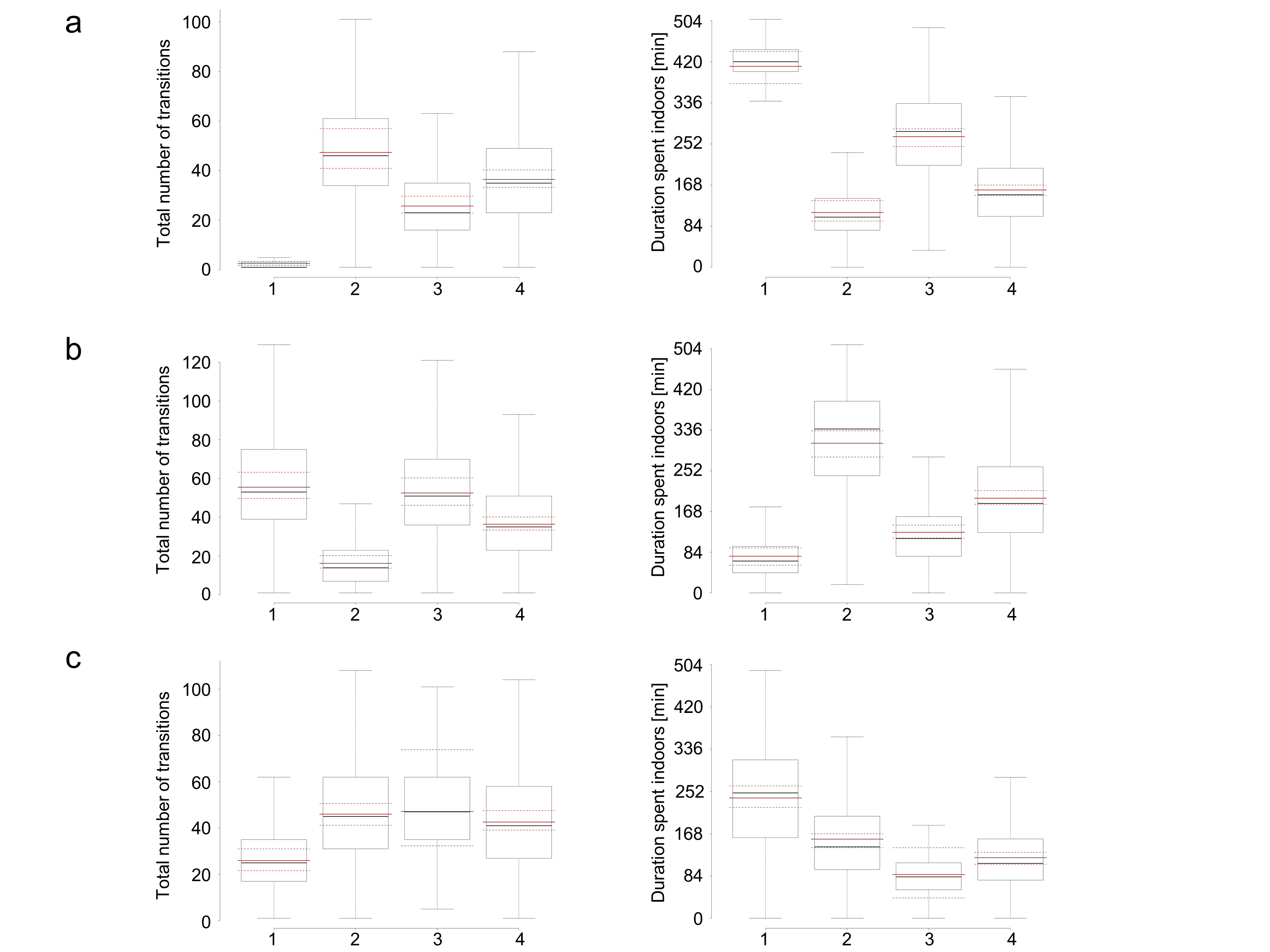

Supplement: Supplementary file 1 [file animals-12-00555-s001.zip › S5 Fig.tiff]

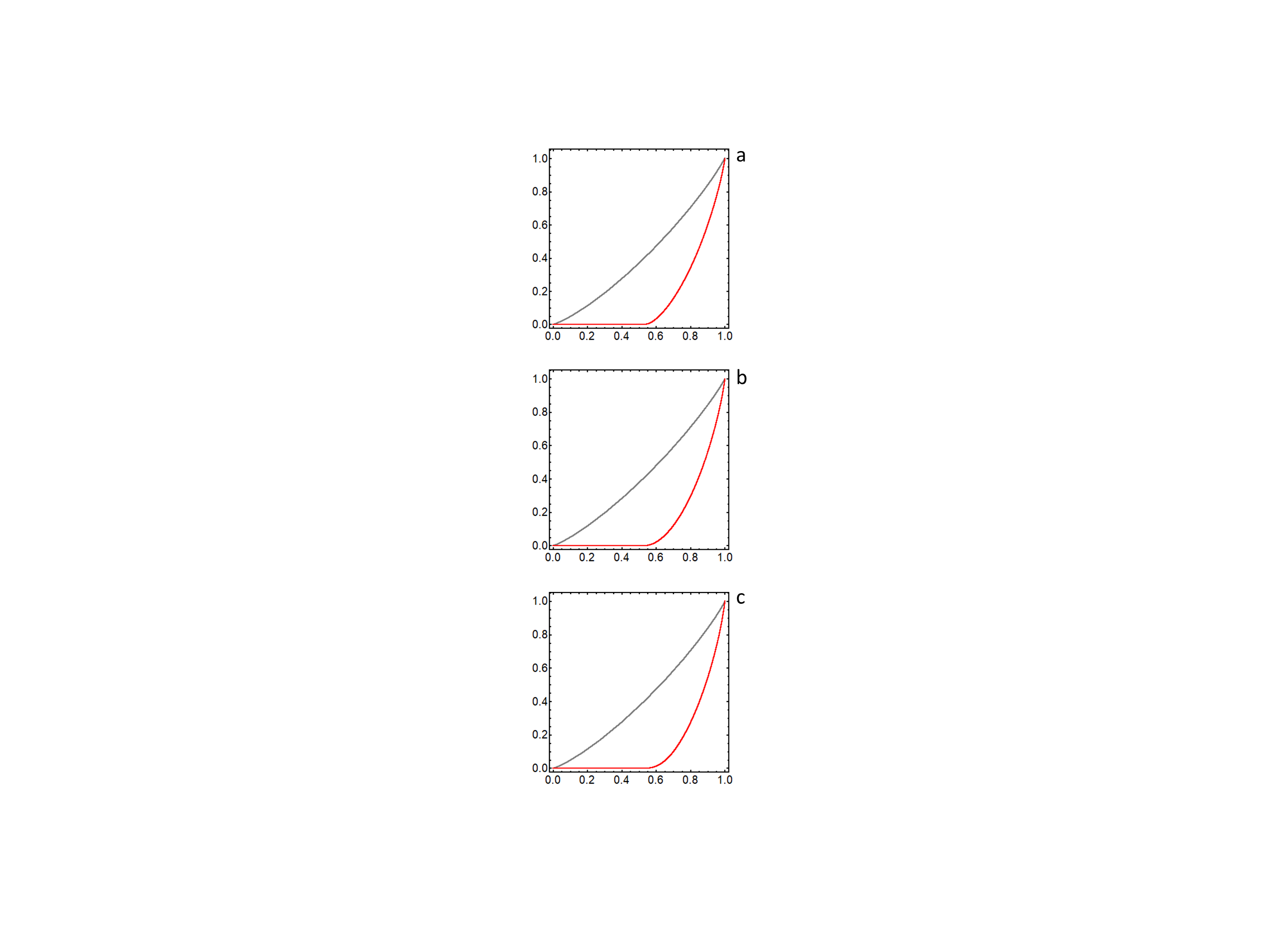

Supplement: Supplementary file 1 [file animals-12-00555-s001.zip › S6 Fig.tiff]

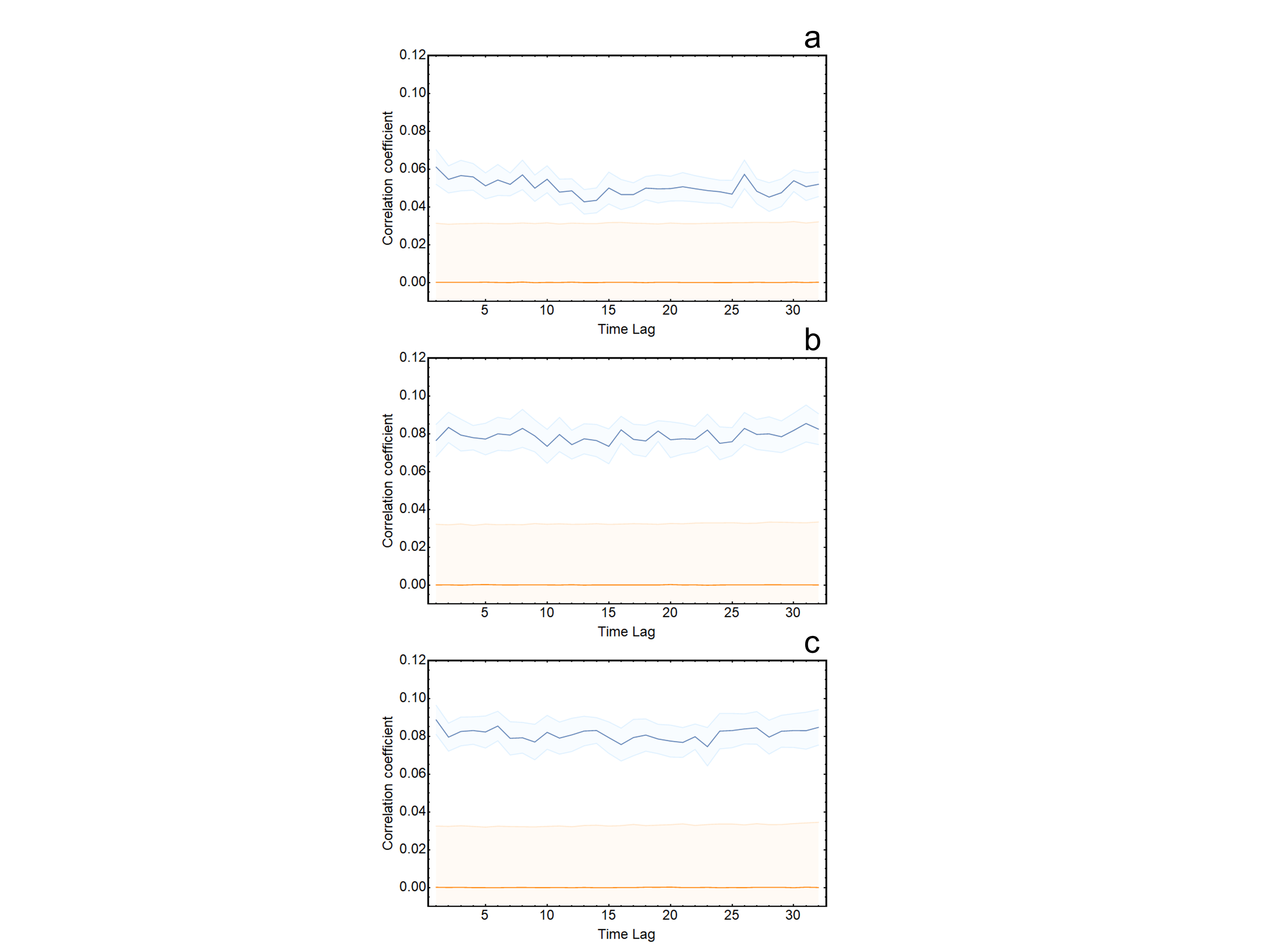

Supplement: Supplementary file 1 [file animals-12-00555-s001.zip › S7 Fig_v3.tiff]

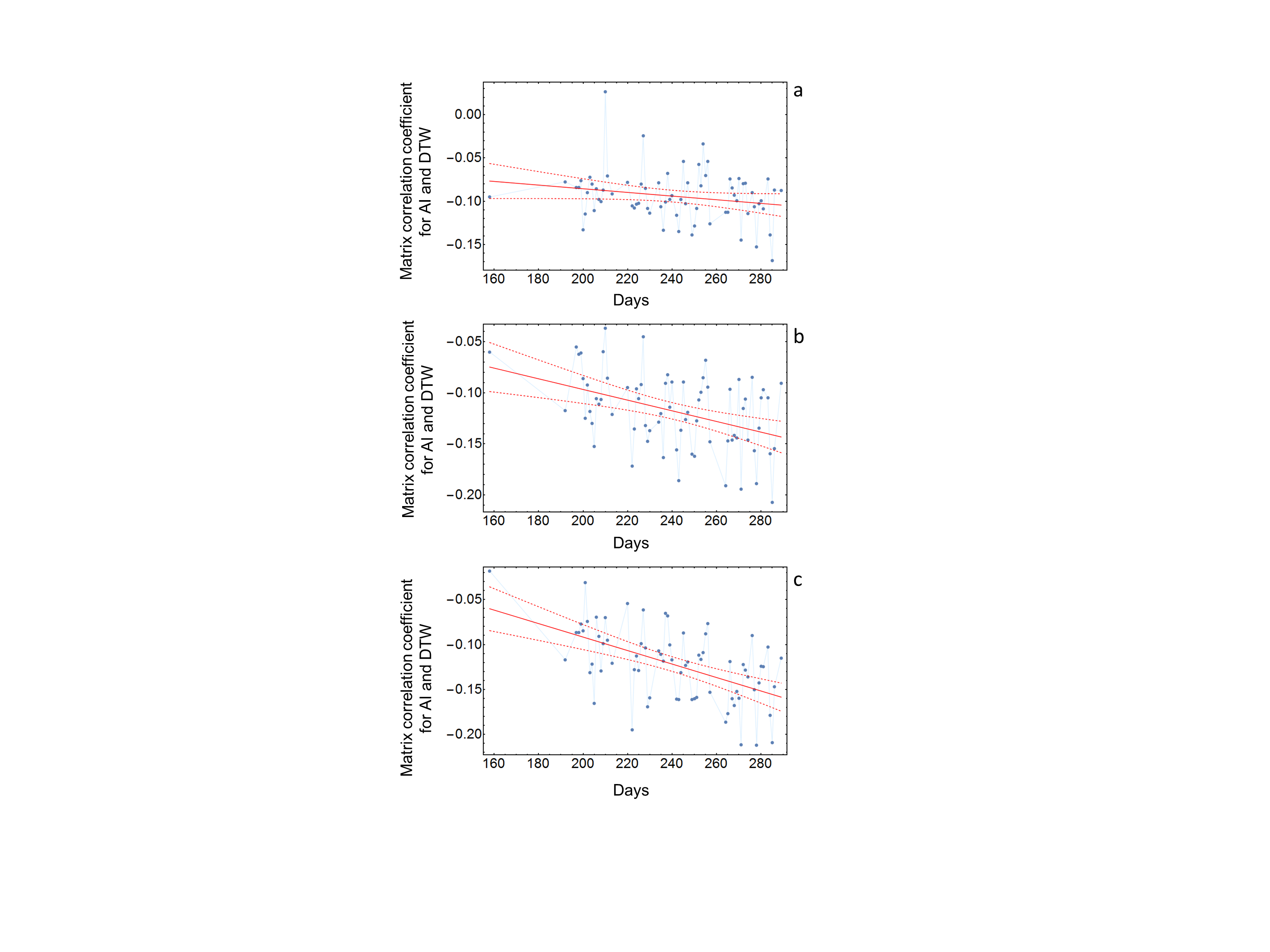

Supplement: Supplementary file 1 [file animals-12-00555-s001.zip › S8 Fig_v3.tiff]

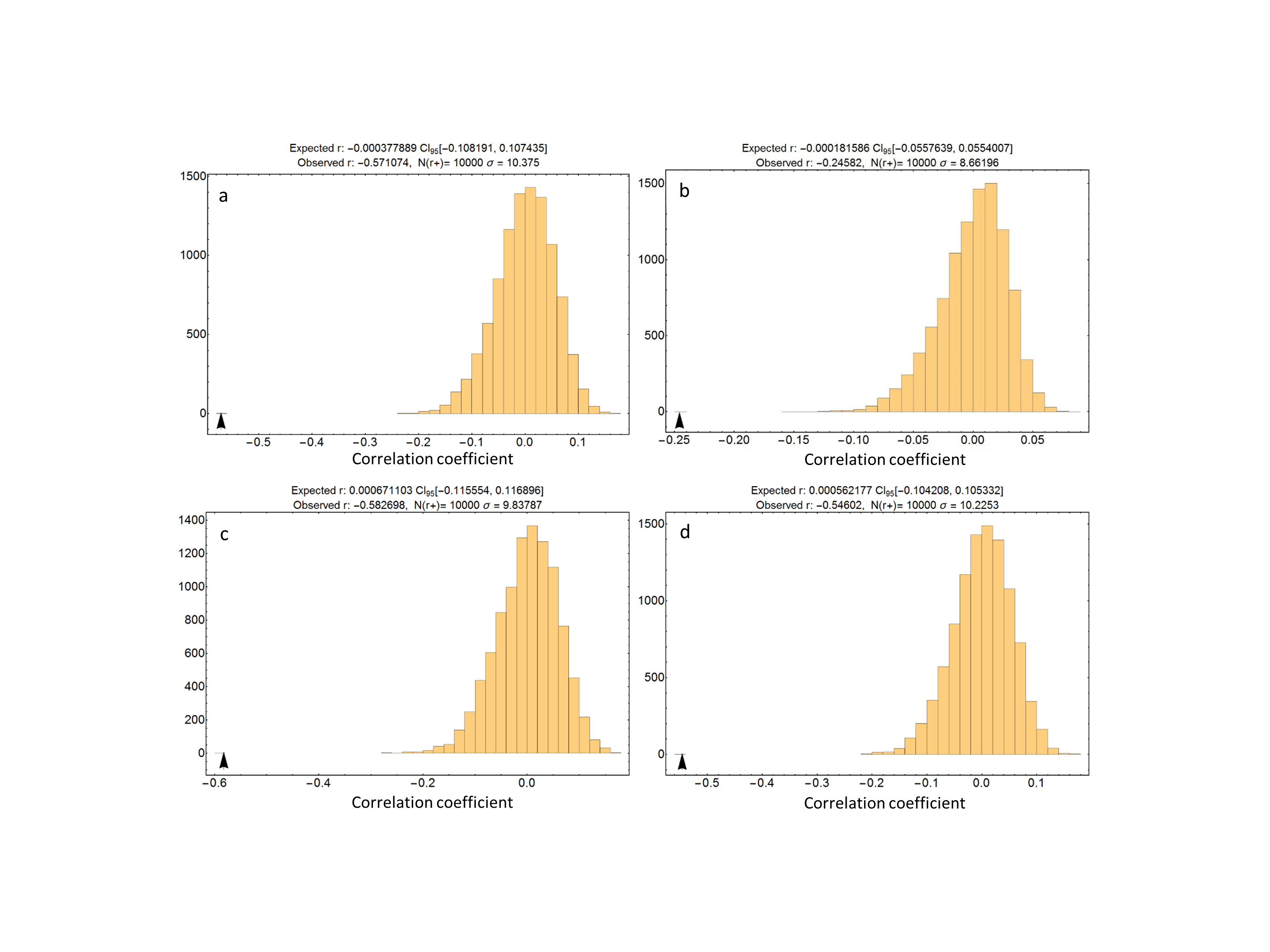

Supplement: Supplementary file 1 [file animals-12-00555-s001.zip › S9 Fig.tif]
